# The frequencies and disparities of adverse childhood experiences in the U.S.

**DOI:** 10.1186/s12889-020-09411-z

**Published:** 2020-09-10

**Authors:** Zachary Giano, Denna L. Wheeler, Randolph D. Hubach

**Affiliations:** Center for Rural Health, Oklahoma State University--Center for Health Sciences, 1111 W 17th Street, Tulsa, OK 74107 USA

**Keywords:** Adverse childhood experiences, BRFSS

## Abstract

**Background:**

Adversity experienced during childhood manifests deleteriously across the lifespan. This study provides updated frequency estimates of ACEs using the most comprehensive and geographically diverse sample to date.

**Methods:**

ACEs data were collected via BRFSS (Behavioral Risk Factor Surveillance System). Data from a total of 211,376 adults across 34 states were analyzed. The ACEs survey is comprised of 8 domains: physical/emotional/sexual abuse, household mental illness, household substance use, household domestic violence, incarcerated household member, and parental separation/divorce. Frequencies were calculated for each domain and summed to derive mean ACE scores. Findings were weighted and stratified by demographic variables. Group differences were assessed by post-estimation F-tests.

**Results:**

Most individuals experienced at least one ACE (57.8%) with 21.5% experiencing 3+ ACEs. F-tests showed females had significantly higher ACEs than males (1.64 to 1.46). Multiracial individuals had a significantly higher ACEs (2.39) than all other races/ethnicities, while White individuals had significantly lower mean ACE scores (1.53) than Black (1.66) or Hispanic (1.63) individuals. The 25-to-34 age group had a significantly higher mean ACE score than any other group (1.98). Generally, those with higher income/educational attainment had lower mean ACE scores than those with lower income/educational attainment. Sexual minority individuals had higher ACEs than straight individuals, with significantly higher ACEs in bisexual individuals (3.01).

**Conclusion:**

Findings highlight that childhood adversity is common across sociodemographic, yet higher in certain categories. Identifying at-risk populations for higher ACEs is essential to improving the health outcomes and attainment across the lifespan.

## Background

Mental and physical health, disease, cognition, well-being, and lifelong health is rooted in childhood. The study of adverse childhood experiences (ACEs) and the panoply of risks associated with these adverse events has grown markedly in the past 20 years [1]. The study of individuals with high ACEs has revealed significant physical health risks such as heart and pulmonary diseases, lung cancer, metabolic issues, inflammation, and liver diseases [2–6]. Mental health is equally affected by ACEs, as studies show strong links to depression, anxiety, severe mood disorders, and suicide [7–10]. Nevertheless, demographic diversity within these studies have been limited, with researchers calling for updated prevalence rates regarding ACEs by demographics and region [11].

Considerable research has been devoted to adverse childhood experiences because of its strong associations to public health issues. Within a public health context, ACEs have been linked to homelessness [12], lifetime alcohol dependence [13], opioid addiction [14], and increased exposure to HIV risk [15, 16]. Though these investigations have been a critical step in the development of health programming to attenuate these outcomes, more recent research regarding ACEs in the context of public health have revealed that while it is generally accepted that ACEs have a cumulative effect, not all populations are equally vulnerable to ACEs [11], and further, certain segments of the population may manifest childhood adversity differently [17] (e.g., one study found that the adverse mental health impact of ACEs on Whites was consistently greater than on Black and Latino individuals), thus suggesting a more complex relationship than traditional linear relationships with ACEs show in the general population. As such, ACEs prevention programming with a public health emphasis has shifted to more tailored-specific programming for specific races/ethnicities and has shown promising results in Black and Hispanic communities [18, 19].

Starting in 2009, the Centers for Disease Control (CDC) gave states the option to collect ACEs data as a part of the Behavioral Risk Factor Surveillance Survey (BRFSS), a national survey of demographics, behaviors, and health indicators [20]. As a product of these surveys, Merrick and colleagues [11] collected the most comprehensive ACEs data to date, acquiring ACEs data from over 200,000 individuals in 23 states from the years 2011 to 2014. Using the same methodology, we have collected ACEs data in the same way.

Compared to Merrick’s and colleagues’ study, our study is methodologically expanded in four important ways that help broaden the depth of ACEs prevalence. The first is that we collected data from 11 additional states that were not included in Merrick et al’s study (a 48% increase). With the additions of New Mexico, West Virginia, Kentucky, Ohio, Texas, Arkansas, Georgia, Hawaii, Louisiana, New York, and Illinois, we believe this expands the breadth of geographic available data on the topic and to our knowledge, is the most current and geographically comprehensive ACEs database to date. In particular, states classified in the South are especially understudied with respect to ACEs. For example, although Merrick and colleagues’ article was the most geographic expansive article to date, their analysis only included 5 of the 16 states in the South (31%), while our investigation includes 12 of the 16 states (75%). This is important due to preliminary data suggesting that southern states may have higher rates of adversity among children compared to other regions [21].

Second, among states already represented in both Merrick’s study and our study, we collected updated data from 13 states (Alaska, Arizona, California, Connecticut, Iowa, Michigan, Nevada, Oklahoma, Oregon, Pennsylvania, South Carolina, Tennessee, and Wisconsin). Third, our study limited data to a single year. Merrick et al. used several years of data for a single state (this was the case for eight states in their study), whereas ours only allows for the latest year of each state counting only once. We believe that using multiple years of the same state possibly inflates the data from that region and also may account for duplicative data (e.g., people may have taken the survey twice and thus were counted twice in their analyses, overrepresentation of a particular state and/or region, unbalanced racial/ethnic categories, etc.). Lastly, Merrick’s study did not utilize any type of significance testing. We further expand on their methods by utilizing post-estimation F-tests to assess differences in ACEs prevalence among demographic variables in order to detect significant differences among groups.

**Fig. 1:**
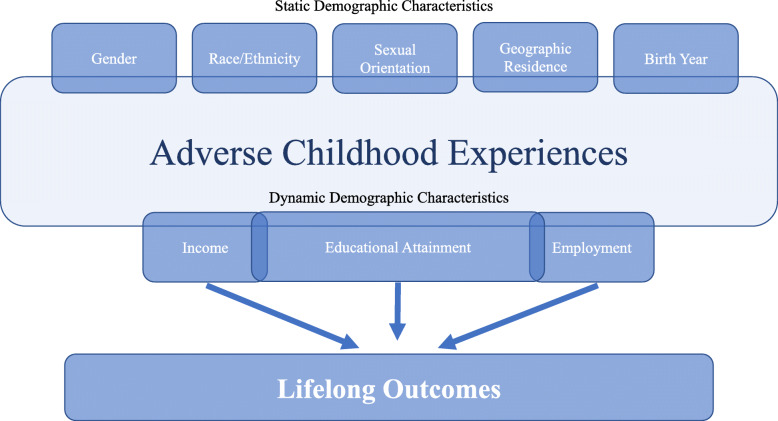
Shows the conceptual framework for demographics characteristics and ACEs

Conceptually, we view the demographic characteristics of individuals bifurcated into two levels which interface with ACEs (see Fig. 1). First, static demographic characteristics are elements which are generally inherent to individuals. These characteristics include gender, race/ethnicity, sexual orientation, geographic residence, and birth year. Next, dynamic demographic differences are traits which change in a more active way beyond childhood, particularly after ACEs. While ACEs have been linked to lifelong outcomes beyond both demographic categories, [1, 22] there may be statistical differences in ACEs by static demographic characteristics, while dynamic demographic characteristics may be influenced by other dynamic demographic characteristics (e.g., education influencing future income), static demographic characteristics (e.g., the effects of gender discrimination on income), and ACEs (e.g., childhood adversity affecting future income) [23, 24]. Moreover, understanding the impact that ACEs has on lifelong outcomes can be better understood by the stratification of individual elements from both categories among ACEs. This is particularly true for the development of prevention/intervention programs centered on ACEs, as programs which are tailored by demographic characteristics have shown greater efficacy [25].

## Methods

Data were obtained from the CDC’s BRFSS; a yearly, national survey that collects data via cellular and landline telephone interviews among adults 18 years and older. The BRFSS uses a multistage sampling design to acquire data on health-related domains from non-institutionalized civilian populations residing in all 50 states, the District of Columbia, and three U.S. territories (Puerto Rico, Guam, and the U.S. Virgin Islands). The BRFSS has three overall modules: 1. core modules are sets of survey questions consistently administered to all states and territories, 2. optional modules which consist of CDC developed questions that states can include in their survey, and 3. state-added questions, which include customizable items developed by each state coordinator. Only the core modules are publicly available. ACEs data was collected as a part of the optional modules. As of 2018, 41 states collected ACE data. It should be noted that several states have collected 2018 data for the first time, however, this data is typically not available until at least 2 years after collection.

The ACEs module consists of 11 questions derived from the CDC’s ACEs study investigating adverse events in childhood before the age of 18 [26]. The survey questions fall into eight adversity domains including emotional abuse, physical abuse, sexual abuse, intimate partner violence (IPV), household substance use, household mental illness, parental separation/divorce, and household members who are incarcerated. The responses were dichotomized and summed, thus creating an ACE score range of 0 to 8 (higher scores indicating greater exposure to adverse events). Ford and colleagues offer an in-depth description of the BRFSS ACE module, factorial structure, and calculated ACE scores [27].

In total, 38 out of 50 states collected ACEs data starting from 2011 to 2017 (Washington D.C. did not collect data). Of these, three states declined to share data for various reasons (e.g., stopped giving data due to lack of resources, data is privately funded and not given publicly, committee declined to have data included in the study, etc.), with one additional state being unresponsive, resulting in a final state count of 34 and a final sample size of 211,376.

**Fig. 2:**
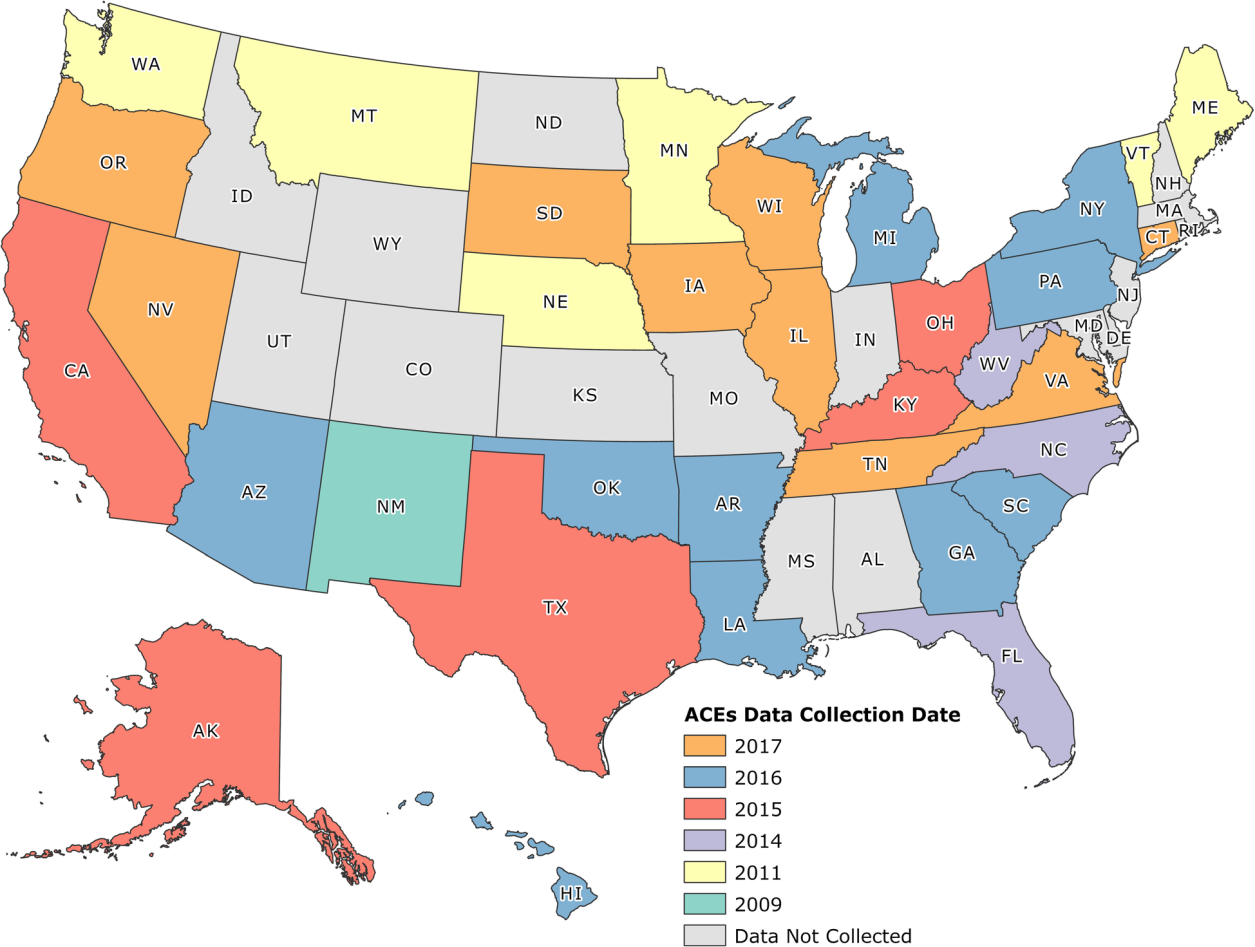
Shows the data collected by state and year, graphically. Image was created by the author team

Following the methodology of Merrick and colleagues [11], states that included ACE items in their optional modules were contacted to establish data use agreements. The ACEs data from each state were merged (along with demographic and weighting variables) from 2009 to 2017 (see Table 1 and Fig. 2). Survey weights, which were included in the acquired data, were used to adjust the data to conform to population parameters provided by the CDC.

**Table 1:** Shows the study breakdown by state and year

**2009**	
New Mexico	
**2011**	
Maine	
Minnesota	
Montana	
Nebraska	
Vermont	
Washington	
**2014**	
Florida	
North Carolina	
West Virginia	
**2015**	
Alaska	
California	
Kentucky	
Ohio	
Texas	
**2016**	
Arizona	
Arkansas	
Georgia	
Hawaii	
Louisiana	
Michigan	
New York	
Oklahoma	
Pennsylvania	
South Carolina	
**2017**	
Connecticut	
Illinois	
Iowa	
Nevada	
Oregon	
South Dakota	
Tennessee	
Virginia	
Wisconsin

### Analytic strategy

First, frequency statistics were computed for the overall sample, males, and females. Estimated weighted frequencies were further stratified by age, race/ethnicity, annual household income, employment status, educational attainment, sexual orientation, and four geographical regions- classified by the U.S. Census Bureau [28]. Next, a frequency analysis was conducted by each of the eight ACE categories using mean ACE scores, stratified by the same sociodemographic variables previously mentioned. Both sets of frequency analyses are weighted with corresponding 95% confidence intervals (CI). Data analyses were conducted using SPSS software, version 24 [29]. Lastly, post-estimation F-tests were used to detect ACEs differences in mean scores (number of ACE exposures) and also differences within types of ACE exposure. We use the method outlined by Cumming [30] in which the 95% confidence intervals of two coefficients are compared. In the event that the confidence intervals overlap by less than half the length of one confidence interval arm, then the *p-*value between the confidence intervals is at least below the level of significance (i.e., less than .05). Previous studies show this method to be sufficiently accurate when two conditions are met: 1. When sample sizes are at least 10, and 2. When the two intervals do not differ in width by more than a factor of 2 [30]. This applies to both dichotomous and continuous variables alike. All comparisons in this study met both assumptions. Further, our sample size is considerably large (in excess of 200,000). Thus, post-estimation F tests are more conservative than traditional group difference tests (e.g., *t-*tests and ANOVAs) and may protect against type 1 errors when analyze a larger sample [30]. It should be noted that this method has been used in other studies which utilized large samples [31, 32].

## Results

### Population frequencies

Table 2 presents the weighted estimates across demographic variables bifurcated by gender (*N* = 211,376). Generally, all age groups were represented, with the lowest percentage being 18 to 24-year-olds (11.5%) and the largest group being 64 and over (21.8%). The majority of the sample was White (65.1%) and identified as heterosexual/straight (95.8%). Approximately one-third of the sample attended some college (31.3%) with the next largest group having a high school degree (28.4%), following by a college degree (25.9%). The majority of individuals were employed (56.5%) had income over $50,000 (47.6%). Additionally, the majority of cases resided in the South (45.4%) followed by the West (20.6%), Midwest (20.1%), and Northeast (13.7%).

**Table 2:** Demographic Characteristics of the Sample

	Male			Female			All		
Characteristic	No.	Wgt %	95% CI	No.	Wgt %	95% CI	No.	Wgt %	95% CI
Age Group									
18–24	4631	12.39	(11.87–12.94)	4268	10.74	(10.25–11.24)	8900	11.53	(11.17–11.9)
25–34	7469	15.61	(15.07–16.17)	9321	14.43	(13.96–14.91)	16,792	14.99	(14.64–15.36)
35–44	9883	16.82	(16.28–17.37)	13,019	15.47	(15.00–15.95)	22,904	16.11	(15.75–16.48)
45–54	14,706	18.09	(17.57–18.62)	20,077	17.66	(17.2–18.14)	34,791	17.87	(17.52–18.22)
55–64	20,365	17.31	(16.86–17.77)	28,207	18.09	(17.66–18.53)	48,575	17.72	(17.41–18.04)
> 64	28,751	19.78	(19.32–20.25)	47,005	23.61	(23.16–24.06)	75,761	21.78	(21.46–22.11)
Race/Ethnicity									
White	67,131	65.17	(64.44–65.88)	95,550	64.98	(64.31–65.63)	162,689	65.06	(64.58–65.55)
Black	4838	10.66	(10.21–11.15)	8689	11.93	(11.5–12.36)	13,528	11.33	(11.01–11.65)
Other	4543	6.31	(5.92–6.72)	5252	5.70	(5.31–6.13)	9798	5.99	(5.71–6.29)
Multiracial	2303	1.45	(1.32–1.59)	3009	1.39	(1.27–1.52)	5314	1.42	(1.33–1.51)
Hispanic	5613	16.41	(15.8–17.04)	7932	16.00	(15.45–16.57)	13,546	16.20	(15.78–16.62)
Household Income									
< 15,000	6179	8.98	(8.55–9.42)	12,326	13.11	(12.64–13.59)	18,505	11.08	(10.76–11.41)
15,000-24,999	10,788	14.97	(14.43–15.52)	18,947	19.03	(18.48–19.59)	29,736	17.04	(16.65–17.43)
25,000-34,999	8101	10.21	(9.78–10.67)	12,130	10.84	(10.44–11.25)	20,236	10.54	(10.24–10.84)
35,000-49,999	11,432	14.22	(13.71–14.76)	15,050	13.33	(12.89–13.79)	26,486	13.77	(13.43–14.12)
> 50,000	39,473	51.62	(50.89–52.36)	42,919	43.69	(43.02–44.36)	82,397	47.57	(47.07–48.07)
Education									
Less than HS	6708	15.12	(14.52–15.73)	9267	13.85	(13.34–14.36)	15,975	14.45	(14.06–14.85)
HS diploma/GED	24,332	29.51	(28.89–30.14)	34,282	27.32	(26.78–27.87)	58,615	28.37	(27.96–28.78)
Some college	21,997	29.54	(28.91–30.18)	35,299	32.89	(32.3–33.49)	57,305	31.29	(30.86–31.73)
College degree	32,570	25.83	(25.32–26.35)	42,783	25.94	(25.46–26.43)	75,362	25.89	(25.54–26.24)
Employment Status									
Employed	47,133	64.70	(64.05–65.34)	52,621	49.01	(48.38–49.63)	99,766	56.50	(56.04–56.95)
Unemployed	4178	5.93	(5.59–6.28)	4805	5.29	(5–5.6)	8984	5.59	(5.37–5.82)
Unable to work	5718	6.88	(6.54–7.24)	10,016	8.41	(8.08–8.76)	15,734	7.68	(7.44–7.93)
Other	27,798	22.49	(21.96–23.03)	53,206	37.29	(36.69–37.88)	81,009	30.23	(29.82–30.64)
Sexual Orientation									
Straight	44,748	96.14	(95.81–96.44)	60,072	95.48	(95.11–95.82)	104,834	95.80	(95.55–96.03)
Gay/Lesbian	935	2.08	(1.86–2.31)	661	1.25	(1.09–1.46)	1596	1.65	(1.52–1.81)
Bisexual	575	1.41	(1.23–1.63)	1000	2.54	(2.28–2.82)	1577	1.99	(1.83–2.17)
Other	167	0.37	(0.28–0.49)	340	0.73	(0.59–0.89)	507	0.56	(0.47–0.65)
Census Region									
Northeast	14,396	13.70	(13.26–14.14)	19,530	13.77	(13.37–14.18)	33,939	13.74	(13.44–14.04)
South	27,665	45.02	(44.34–45.71)	42,265	45.78	(45.16–46.39)	69,931	45.41	(44.96–45.87)
Midwest	21,205	20.26	(19.76–20.77)	29,748	20.17	(19.72–20.63)	50,958	20.22	(19.88–20.56)
West	22,539	21.02	(20.43–21.61)	30,354	20.28	(19.73–20.84)	52,895	20.63	(20.23–21.04)

### Prevalence of ACEs

In total, the majority of individuals experienced at least one adverse experience (57.8%). Approximately 42% had an ACE score of 0, followed by 22.9% (1 ACE), 12.8% (2 ACEs), 8.2% (3 ACEs), 5.7% (4 ACEs), 3.8% (5 ACEs), 2.3% (6 ACEs), 1.2% (7 ACEs), and 0.3% (all 8 ACEs; not shown in tables). Table 3 presents the prevalence of ACEs by demographic variables among all eight ACE categories, as well as a total ACE mean score.

**Table 3:** Frequencies of ACE Types and Mean ACE Score by Sociodemographic Characteristics (Weighted Percent and 95% CI)

	Emotional	Physical	Sexual	IPV	House Sub. Abuse	Household Mental Ill.	Parental Sep/Div.	Incarcerated Member	ACE Score Mean	Mean Score Sig. Diff.
Total	33.46	17.50	11.31	17.76	26.83	16.16	28.24	8.08	1.56	
	(33.02–33.91)	(17.14–17.87)	(11.02–11.60)	(17.40–18.13)	(26.42–27.23)	(15.82–16.50)	(27.81–28.67)	(7.81–8.36)	(1.54–1.57)	
Sex										
1. Male	33.57	17.46	6.18	17.00	25.58	13.59	27.84	8.47	1.46	2 > 1
	(32.91–34.25)	(16.92–18.02)	(5.87–6.51)	(16.46–17.56)	(24.98–26.19)	(13.11–14.08)	(27.20–28.48)	(8.06–8.90)	(1.44–1.49)	
2. Female	33.37	17.54	16.05	18.46	27.97	18.50	28.60	7.73	1.64	
	(32.77–33.97)	(17.06–18.04)	(15.59–16.51)	(17.97–18.95)	(27.42–28.52)	(18.03–18.99)	(28.02–29.19)	(7.38–8.09)	(1.62–1.67)	
Age Group										
1. 18–24	42.02	18.32	9.67	17.26	28.29	24.43	39.33	16.30	1.91	2 > all
	(40.29–43.76)	(17.00–19.72)	(8.71–10.73)	(16.01–18.57)	(26.80–29.82)	(23.02–25.91)	(37.64–41.05)	(15.07–17.61)	(1.84–1.97)	1 > 3,4,5,6
2. 25–34	40.05	21.07	11.42	21.71	31.52	23.05	40.72	14.10	1.98	3 > 4,5,6
	(38.73–41.39)	(19.95–22.24)	(10.61–12.27)	(20.59–22.88)	(30.30–32.78)	(21.96–24.18)	(39.39–42.06)	(13.17–15.07)	(1.93–2.04)	4 > 5,6
3. 35–44	36.65	20.31	13.56	20.99	29.67	18.21	35.04	9.26	1.79	5 > 6
	(35.46–37.85)	(19.31–21.35)	(12.75–14.42)	(19.98–22.05)	(28.57–30.79)	(17.32–19.15)	(33.86–36.23)	(8.57–9.99)	(1.74–1.84)	
4. 45–54	35.69	19.11	13.62	19.11	28.08	15.52	29.07	6.44	1.63	
	(34.65–36.74)	(18.26–19.99)	(12.92–14.35)	(18.27–19.98)	(27.14–29.04)	(14.80–16.28)	(28.09–30.07)	(5.92–7.01)	(1.59–1.67)	
5. 55–64	32.75	17.03	12.07	18.28	27.61	13.92	20.50	5.05	1.44	
	(31.85–33.66)	(16.31–17.79)	(11.47–12.69)	(17.53–19.05)	(26.79–28.45)	(13.30–14.56)	(19.73–21.28)	(4.63–5.51)	(1.41–1.47)	
6. > 64	20.80	11.63	7.91	11.41	19.08	7.88	14.82	2.58	0.94	
	(20.18–21.43)	(11.14–12.14)	(7.54–8.31)	(10.92–11.92)	(18.49–19.68)	(7.51–8.26)	(14.26–15.39)	(2.37–2.82)	(0.92–0.96)	
Race/Ethnicity										
1. White	34.01	16.35	11.23	15.95	27.62	18.18	26.00	6.67	1.53	4 > all
	(33.52–34.15)	(15.97–16.74)	(10.91–11.55)	(15.57–16.34)	(27.17–28.08)	(17.78–18.59)	(25.53–26.48)	(6.39–6.95)	(1.51–1.54)	2 > 1,3
2. Black	29.97	13.46	12.30	20.88	25.87	12.22	43.84	14.45	1.66	5 > 1,3
	(28.56–31.41)	(12.42–14.57)	(11.34–13.33)	(19.65–22.16)	(24.57–27.21)	(11.19–13.33)	(42.29–45.41)	(13.37–15.60)	(1.60–1.71)	1 > 3
3. Other	29.60	18.02	8.01	16.51	15.30	11.10	17.89	5.62	1.18	
	(27.35–31.95)	(16.19–20.02)	(6.89–9.30)	(14.81–18.36)	(13.73–17.03)	(9.78–12.56)	(16.15–19.77)	(4.68–6.75)	(1.10–1.25)	
4. Multiracial	47.12	27.10	19.29	27.26	39.31	26.78	44.60	14.95	2.39	
	(43.90–50.37)	(24.35–30.04)	(16.88–21.96)	(24.52–30.19)	(36.25–42.45)	(24.04–29.71)	(41.41–47.83)	(12.77–17.42)	(2.26–2.51)	
5. Hispanic	33.84	23.70	11.34	22.40	27.07	11.55	29.30	9.35	1.63	
	(32.43–35.28)	(22.45–24.99)	(10.46–12.28)	(21.19–23.67)	(25.80–28.38)	(10.64–12.51)	(27.98–30.67)	(8.51–10.27)	(1.58–1.69)	
Household Income										
1. < 15,000	37.70	25.16	16.31	25.06	33.89	19.33	36.73	12.64	2.00	1 > all
	(36.17–39.25)	(23.80–26.57)	(15.21–17.47)	(23.70–26.46)	(32.44–35.38)	(18.13–20.60)	(35.19–38.30)	(11.61–13.74)	(1.97–2.06)	2 > 3,4,5
2. 15,000-24,999	33.95	20.17	13.61	22.11	29.26	16.32	32.05	10.92	1.73	3 > 5
	(32.73–35.19)	(19.13–21.25)	(12.77–14.49)	(21.00–23.26)	(28.13–30.42)	(15.41–17.28)	(30.83–33.29)	(10.08–11.82)	(1.68–1.78)	4 > 5
3. 25,000-34,999	33.21	18.79	12.00	19.49	27.84	15.17	28.23	8.85	1.59	
	(31.75–34.70)	(17.57–20.08)	(11.07–12.99)	(18.23–20.80)	(26.50–29.22)	(14.10–16.31)	(26.85–29.65)	(7.99–9.79)	(1.54–1.65)	
4. 35,000-49,999	35.03	18.08	11.83	18.90	27.45	15.83	29.49	8.88	1.62	
	(33.71–36.37)	(17.00–19.21)	(10.98–12.73)	(17.82–20.02)	(26.28–28.66)	(14.87–16.83)	(28.22–30.80)	(8.07–9.77)	(1.56–1.67)	
5. > 50,000	33.76	15.48	9.83	15.27	25.24	16.80	25.13	5.77	1.44	
	(33.09–34.44)	(14.96–16.02)	(9.43–10.25)	(14.76–15.79)	(24.64–25.85)	(16.27–17.34)	(24.50–25.77)	(5.42–6.13)	(1.42–1.47)	
Educational Attainment										
1. Less than HS	31.83	22.87	12.22	23.49	30.92	13.01	31.55	10.90	1.71	1 > 2,4
	(30.40–33.30)	(21.59–24.21)	(11.26–13.24)	(22.20–24.83)	(29.55–32.32)	(12.02–14.06)	(30.13–33.00)	(9.99–11.89)	(1.66–1.77)	3 > 2,4
2. HS diploma/GED	32.12	17.37	10.43	18.21	27.55	14.73	31.17	9.94	1.57	2 > 4
	(31.30–32.96)	(16.69–18.06)	(9.94–10.95)	(17.53–18.91)	(26.79–28.32)	(14.10–15.38)	(30.35–32.00)	(9.37–10.54)	(1.54–1.61)	
3. Some college	36.88	18.42	13.38	18.74	29.08	18.85	30.80	8.56	1.70	
	(36.04–37.72)	(17.75–19.10)	(12.82–13.96)	(18.08–19.43)	(28.32–29.84)	(18.19–19.53)	(29.99–31.63)	(8.08–9.06)	(1.67–1.73)	
4. College degree	31.82	13.61	9.36	13.03	21.11	16.25	20.24	3.95	1.26	
	(31.14–32.50)	(13.12–14.11)	(8.97–9.76)	(12.55–13.52)	(20.56–21.68)	(15.74–16.77)	(19.67–20.83)	(3.68–4.23)	(1.24–1.29)	
Employment Status										
1. Employed	35.64	17.41	10.81	18.22	27.39	17.02	30.51	8.55	1.61	3 > all
	(35.02–36.26)	(16.91–17.92)	(10.43–11.21)	(17.71–18.74)	(26.83–27.96)	(16.55–17.50)	(29.91–31.11)	(8.17–8.93)	(1.59–1.64)	
2. Unemployed	39.42	24.80	15.39	23.04	34.10	20.77	39.99	14.39	2.05	2 > 1,4
	(37.39–41.50)	(22.97–26.72)	(13.99–16.89)	(21.33–24.84)	(32.17–36.09)	(19.16–22.47)	(37.91–42.11)	(12.84–16.09)	(1.97–2.14)	1 > 4
3. Unable to work	41.20	27.09	22.19	27.74	40.07	23.73	36.43	13.22	2.24	
	(39.55–42.87)	(25.61–28.63)	(20.77–23.67)	(26.24–29.30)	(38.46–17.71)	(22.38–25.13)	(34.79–38.09)	(12.08–14.46)	(2.17–2.31)	
4. Other	27.08	14.06	9.16	13.80	21.52	12.37	20.43	5.17	1.20	
	(26.34–27.83)	(13.49–14.65)	(8.74–9.61)	(13.25–14.38)	(20.89–22.17)	(11.83–12.93)	(19.75–21.12)	(4.79–5.59)	(1.18–1.23)	
Sexual Orientation										
1. Straight	33.76	17.59	10.54	17.34	26.20	15.42	27.89	7.64	1.53	
	(33.19–34.33)	(17.13–18.06)	(10.20–10.89)	(16.88–17.80)	(25.70–26.72)	(15.00–15.85)	(27.35–28.44)	(7.31–7.98)	(1.51–1.55)	
2. Gay/Lesbian	48.05	28.77	23.60	27.68	36.73	26.31	33.41	12.13	2.30	3 > all
	(43.53–52.60)	(24.85–33.05)	(19.90–27.75)	(24.00–31.69)	(32.54–41.13)	(22.78–30.18)	(29.41–37.67)	(9.43–15.47)	(2.11–2.49)	2 > 1,4
3. Bisexual	58.32	35.03	30.97	27.60	46.62	44.05	43.17	21.49	3.01	1 > 4
	(53.95–62.56)	(30.96–39.33)	(27.18–35.03)	(24.01–31.51)	(42.37–50.93)	(39.79–48.39)	(38.88–47.57)	(18.13–25.28)	(2.83–3.20)	
4. Other	34.75	21.73	9.68	17.45	24.66	13.72	25.47	6.58	1.50	
	(27.61–42.64)	(16.16–28.58)	(6.72–13.74)	(12.39–24.01)	(19.01–31.33)	(9.89–18.71)	(19.33–32.77)	(3.67–11.53)	(1.22–1.79)	
Census Region										
1. Midwest	35.96	16.95	11.71	16.68	26.53	17.08	25.12	7.92	1.56	4 > all
	(35.07–36.86)	(16.24–17.68)	(11.12–12.32)	(15.99–17.40)	(25.71–17.36)	(16.36–17.82)	(24.28–25.98)	(7.36–8.51)	(1.52–1.59)	
2. Northeast	34.72	17.08	10.54	15.89	26.00	17.40	25.24	6.92	1.52	
	(33.61–35.84)	(16.22–17.97)	(9.85–11.26)	(15.04–16.78)	(25.00–27.03)	(16.51–18.34)	(24.20–26.31)	(6.31–7.58)	(1.47–1.56)	
3. South	29.85	15.96	11.42	17.83	25.99	15.60	29.55	8.23	1.49	
	(29.21–30.51)	(15.42–16.51)	(10.98–11.87)	(17.27–18.40)	(25.39–26.59)	(15.10–16.12)	(28.89–30.22)	(7.82–8.66)	(1.47–1.52)	
4. West	38.52	21.71	11.22	19.94	29.52	15.64	30.41	8.70	1.70	
	(39.36–39.70)	(20.79–22.66)	(10.57–11.91)	(19.05–20.85)	(28.52–30.53)	(14.89–16.41)	(29.39–31.45)	(8.08–9.36)	(1.66–1.74)	

Note: *IPV* Interpersonal Violence

#### ACE domains

Overall, the most common type of ACE domain was emotional abuse (33.5%), followed by parental separation/divorce (28.2%), household substance abuse (26.8%), IPV (17.8%), physical abuse (17.5%), household mental illness (16.2%), sexual abuse (11.3%), and incarcerated household member (8.1%). The frequency of each ACE domain significantly differed from all other categories except for the prevalence between IPV and physical abuse.

#### Gender

Post-estimation F-tests revealed that females had a significantly higher ACE score compared to males (1.64 to 1.46). Females had a significantly higher prevalence of adverse events in four of the eight categories (sexual, IPV, household substance abuse, and household mental illness), while males had a significantly higher prevalence of an incarcerated household member. No significant differences were found in emotional, physical, or divorce categories.

#### Age

F-tests showed that the 25 to 34 age group had a significantly higher ACE mean score than any other group (1.98), while the 64 and over group had a significantly lower ACE mean score than all other groups (0.94). With the exception of the 18 to 24 group compared to the 25 to 34 group, all groups differed significantly from one another. Of note, large disparities were found between the groups of 18 to 24 and 25 to 34 compared to all other older age groups in the categories of incarcerated household member and household mental illness.

#### Race/ethnicity

Individuals who identified as Multiracial had a significantly higher ACE mean score than all other races/ethnicities. This was also true for Multiracial individuals in six of the eight categories (emotional, physical, sexual, IPV, household substance abuse, and household mental illness). Individuals identifying as White had significantly lower mean ACE scores than those identifying as Black or Hispanic, while the “other” category had a significantly lower mean ACE score than all other categories.

#### Household income

Those making less than $15,000 per year had a significantly higher mean ACE score compared to all other categories. This group also had a significantly higher prevalence than all other groups in each of the eight categories. The $15,000 to $24,999 group had significantly higher mean ACE scores than all higher earning groups, while the $50,000+ category had significantly lower mean ACE scores compared to all groups.

#### Educational attainment

Individuals that earned a college degree had a significantly lower mean ACE score compared to all other groups. This was also true for ACE prevalence in six of the eight categories (physical, sexual, IPV, household substance abuse, divorce, and incarcerated household member). Those who earned less than a high school degree had a significantly higher prevalence of adversity in physical, IPV, and household substance abuse compared to all other categories.

#### Employment status

Those in the unable to work category had a significantly higher mean ACE score than all other employment categories (as well as six of the eight individual ACE categories), while those in the other category (including retirees, students, and homemakers) had a significantly lower mean ACE score than all other employment categories (also true in each of the eight individual ACE categories). It should be noted that the unemployed category had a significantly higher mean ACE score than those who were employed.

#### Sexual orientation

Bisexual individuals had a significantly higher prevalence of adversity in seven of eight categories (the exception being IPV) as well as high mean ACE scores. Of particular note, approximately 58% of bisexual individuals reported adversity in the emotional abuse category, the single highest percent of any adversity category across all groups in Table 3. Gay and lesbian individuals had significantly higher mean ACE scores than straight or “other” individuals.

#### Census region

Those residing in the West had a significantly higher mean ACE score compared to the other three regions (as well as four of the eight adversity categories including emotional, physical, IPV, and household substance abuse).

## Discussion

The current study, to our knowledge, is the most diverse and comprehensive compilation of ACEs data and provides an expanded investigation of ACEs exposure across 34 states. Similar to Merrick and colleagues [11], our findings reveal ACEs are prevalent across all demographic variables. There are, however, some populations that experience higher rates of adversity compared to others. In particular, four categories showed particular vulnerabilities to ACEs: females, younger adults, sexual minorities, and multiracial individuals.

While the confidence intervals for females overlapped with the confidence intervals in males in seven of the eight categories, there was a substantial difference (and no confidence interval overlap) between the frequencies of sexual assault for females compared to males (16 to 6%, respectively). Though this difference in stark, it seems unsurprising given past research has shown that while one in five women experience sexual assault, only one out of 70 men experience sexual assault [33], thus accounting for an overall higher mean ACE score in females. Generally, those who were younger reported higher mean ACEs than older individuals. Three possible rationales exist for these disparities. The first is that research has suggested that ACEs may be increasing [34]. Next, it is possible that individuals with higher ACEs may experience early death (thus these individuals are not representative in the data), as empirical evidence claims strong linkages between ACEs and shortened lifespans [35]. Lastly, it is possible that older individuals tend to minimize and/or fail to recall adverse childhood events [22], though this is less extensively studied in the literature. Additionally, there is currently a greater emphasis on/recognition of mental health issues when compared to past decades, thus creating the possibility that older individuals may not have perceived past events as markers of certain ACEs, such as familial mental illness.

With respect to individuals who identify as a sexual minority, our findings are similar to other studies which found a higher prevalence of adverse events among gay and bisexual individuals [36]. Though theories about why sexual minorities have higher ACEs have been postulated, such as certain types of abuse may catalyze shifts in sexual orientation or that sexual minorities may be more likely to recognize, and thus report, adverse events [36], the association of higher ACEs and sexual minorities remains unclear. Lastly, individuals who identified as multiracial had higher frequencies of ACEs than other races/ethnicities, though it should be noted that the frequencies in certain categories mirrored the frequencies of Black and/or Hispanic individuals. This aligns with other research highlighting that social and structural factors elevate the risk of childhood adversity, and that identifying as a racial/ethnic minority creates unique family stress that catalyzes adverse events [11, 37].

Consistent with past research on ACEs, there were notable differences in mean ACE scores in the socioeconomic categories of education, income, and employment [11]. Income generally had a linear relationship with ACEs (i.e., greater income was associated with lower mean ACE scores), with the exception that making $35,000 to $49,999 had a higher mean ACE score than those making $25,000 to $34,000, though the difference was not statistically significant.

With regard to education, there was no significant difference between less than high school and some college, while having a high school degree were associated with significantly lower ACEs. Having a college degree was significantly associated with the lowest ACEs. Data from the 2016 census revealed that more individuals have college degrees now more than ever, [38] and as such, it may be that the first three categories of education (all below having a college degree) represent a lower level of attainment compared to past decades where differences in these categories were more delineated- thus possibly explaining why there was no significant difference in having some college and not completing high school. It is also possible that some individuals were not old enough in order to complete a college degree, and thus, the data may be slightly skewed with respect to educational attainment; although it should be noted that the 18 to 24 age group only comprised 4% of the total sample.

With respect to employment status, those who were unemployed or out of work had high mean ACE scores than those who were employed. Because ACEs are associated with higher rates of disease and injury, it is possible that those with higher ACEs were unable to work due to a physical or mental ailment which impaired their ability to seek employment.

There is also a strong argument to be made regarding the impact that individual ACE domains have. Our results showed that emotional abuse was the most prevalent (33%), while sexual abuse was the least prevalent (11%), however, research has shown that these domains do not have equitable effects [39]. As such, the prevalence of ACEs domains should not be confused with correlations of impact (i.e., emotional abuse treated as a bigger issue than sexual abuse solely because of increased prevalence), particularly as programming is developed to limit childhood adversity and long-term sequelae.

## Conclusions

Our study should be considered in conjunction with several limiting factors. As with all cross-sectional studies, causal inferences should not be taken as sacrosanct, as longitudinal data on adversity exposure is necessary. Next, although previous studies have established acceptable validity of self-reported adversity in childhood [40], the BRFSS relies on data that is self-reported, and thus, may be susceptible to memory and response biases [41]. Additionally, adversity is a complex, multi-dimensional set of processes that the ACEs framework attempts to simplify. There is an argument to be made that not all ACE categories are equal [42], and that protective processes may be just as important as adverse conditions across the lifespan [43]. The BRFSS does not account for multiple instances of a single adversity category (e.g., multiple instances of sexual abuse may be cumulatively as detrimental as experiencing adversity in multiple categories). It should also be noted that that the traditional ACE measure used in this study may not accurately reflect adversity experiences (particularly for individuals identifying as a racial/ethnic minority), and as such, calls for a more nuanced expansion of ACEs have been made [44].

Despite these limitations, our study has several implications for population-based public health. In particular, our study comprises the most comprehensive published ACEs dataset, which captures disparities across a broader geographic spectrum. This would be particularly helpful in a targeted campaign for specific demographic groups to help prevent ACEs. Nevertheless, while the prevention of ACEs is a complicated and difficult public health initiative, there is evidence to suggest that resilience and intervention programming for children aged 6 to 17 can help attenuate the deleterious effect of ACEs among children already experiencing adversity, [45] while protective factors (e.g., an adult who made a child feel safe and protected) have been shown to mitigate the effects of ACEs [46]. These programs would benefit from understanding ACEs from a population-based perspective, thus tailoring programs to those in high risk categories.

## Data Availability

Original data was obtained from the Centers for Disease Control and Prevention’s (CDC) Behavioral Risk Factor Surveillance System (BRFSS). Data are not available due to data sharing agreements with individual states.

## Abbreviations

ACEs: Adverse Childhood Experiences
IPV: Interpersonal Violence
BRFSS: Behavioral Risk Factor Surveillance System
